# Supplementary material for: BUSZ: compressed BUS files
Source: Bioinformatics. 2023 May 2;39(5):btad295. doi: 10.1093/bioinformatics/btad295 (PMC10185401; doi:10.1093/bioinformatics/btad295)
Supplement: btad295_Supplementary_Data [file btad295_supplementary_data.zip › Compressing_BUS_files_supplementary_material.pdf]

BUSZ: Compressed BUS files - Supplement

April 21, 2023

1 Comparing BUStools with other methods

We compared BUStools compression (version 0.42.0) with gzip version 1.6 and zstd version 1.5.2 in a miniconda environment using Python version 3.6.9 and Snakemake version 7.14.2 on a machine running Ubuntu 18.04.6 LTS and Linux kernel 4.15.0-196-generic x86_64. We used GNU time version 1.7 for timing the compression and decompression. For fairness, we cached data files before compression and decompression for each tool, using `dd` coreutil version 8.28. The following snippet shows how a BUS file is compressed and decompressed using BUStools. It also shows the command we used for caching when benchmarking the methods. The full benchmarking workflow can be seen in the Snakefile in the accompanying GitHub repository, https://github.com/pmelsted/BUSZ_paper.

```
# Cache and compress input.bus
dd if=input.bus of=/dev/null bs=1M && \
bustools compress -o output.busz input.bus

# Cache and decompress output.busz
dd if=output.busz of=/dev/null bs=1M && \
bustools inflate -o inflated.bus output.busz
```

2 Supplementary data

The input data was a barcode corrected and sorted BUS file from each experiment considered in (Booeshaghi *et al.*, 2022) labeled by a corresponding accession number. The supplementary dataset contains the result of all experiments performed with information about the compression method, accession number (`sample_id`), original file size, compressed size, compression time, and decompression time.

Table 1: Comparison of the three methods with various compression levels. Note that the speed of BUStools is the same order of magnitude as zstd.

Method	Compression speed (MB/s)	Decompression speed (MB/s)	Compression ratio
`bus`	194.33	275.53	8.29
`gzip -1`	90.04	175.15	4.86
`gzip -9`	3.51	176.84	5.41
`zstd -1`	262.29	402.39	5.29
`zstd -3`	222.68	438.28	5.53
`zstd -19`	1.54	323.18	5.84

3 Fibonacci Encoding

As Fibonacci encoding does not guarantee a byte-aligned encoding, we pad a Fibonacci-encoded block with 0-bits such that the number of bytes for that block is a multiple of 4 or 8, depending on the type of integer used for encoding (`uint32_t` or `uint64_t`).

4 Compressing the Equivalence Class column

4.1 NewPFD

To compress the EC column, we use a compression scheme (*codec*) called NewPFD. This codec is based on another codec called PFOR Delta (PFD), which in turn is a variant of Patched Frame of Reference (PFOR) with delta encoding, i.e. differences. Since the ECs are not sorted, we skip computing the differences.

The PFD codec compresses lists of integers by splitting the input into chunks of e.g. 128 numbers, and has parameters b, and k, such that a certain fraction of numbers, p, fit in the interval $[k, k+2^b-2]$. Any number not in the interval is considered an exception. Let B be a block of numbers to encode. To encode B, we allocate $b * |B|$ bits as a primary encoding block; B_p. Each number $x \in B$ is encoded in B_p using exactly b bits if it fits the interval defined by b and k. Exceptions are indicated with b 1-bits in B_p and their values are encoded after B_p using any codec, e.g. Fibonacci encoding or Elias Delta encoding.

In NewPFD, we store the lower b bits of the exceptions in B_p. The exceptions are then stored in two additional vectors. The former vector stores the gaps between the indices of exceptions in B_p, whereas the latter stores the overflowing high bits. These vectors can then be compressed by any codec. This allows the defined interval above to be $[k, k+2^k-1]$.

Our implementation of NewPFD encodes the parameters, number of exceptions, along with the two exception vectors using Fibonacci encoding prior to the encoding of B_p. This is due to the padding we described in Section 3. For the Fibonacci encoding, we use `uint32_t` to save bits lost due to padding.

4.2 Other methods

We tested several other methods to compress the ECs. These methods were composed of zero or more list encoders, followed by an integer encoder. We define a stack as a compression scheme with multiple layers of encoders. For each of the following stacks, we tested compression with one of several integer encoders. Each layer in the stack, save the first, is applied to the output of the previous stack.

- *empty*: no compression prior to integer encoders.
- **Xor - Runlength**: Runlengh-encoding applied to the XORed values.
- **Xor**: Adjacent values are XORed together.
- **Xor - Delta - Runlength**: Run length encoding applied to the output of **XOR - differences**.
- **Xor - Delta**: Differences (delta) applied to the XORed values.

The following integer encoders were tested:

- **Elias Delta**
- **Elias Delta Rice**, $k \in \{2, 4, 8, 16, 24, 32\}$. Written EliasRiceDelta_$\langle k \rangle$ in the Table 2.
- **Exponential Golomb**, $k \in \{2, 3, 5, 8, 13, 21\}$. Written as ExpGolomb_$\langle k \rangle$_$Enc_H$_$Enc_R$ where Enc_H and Enc_R are the encoders used to encode the variables H and R, respectively. Refer to Section 4.4 for the description of this method. Here we let Enc_H be unary encoding and Enc_R be binary encoding using $H + k$ bits.
- **Exponential Elias**, $k \in \{2, 3, 5, 8, 13, 21\}$, denoted by ExpElias_$\langle k \rangle$ in Table 2.
- **Fibonacci encoding**
- **Huffman encoding**

Some encoders require that the input domain is semi-positive or strictly positive. Thus, we define the following transformations to transform the values to the applicable domain:

- **Increment**: increments the input by one.
- **NegToPosMap**: $\mathbb{Z} \mapsto \mathbb{Z}_+ \cup \{0\}$ with

$$\text{NegToPosMap}(z) = \begin{cases} 2z, & \text{if } z \geq 0 \\ -2z-1, & \text{otherwise.} \end{cases}$$

Exponential Elias uses the same decomposition of an integer into a bucket and an offset as Exponential Golomb, but encodes them using Elias Delta encoding, instead of unary encoding and fixed-width binary encoding.

In addition to the stacks created by the cross product of the above lists, we tested NewPFD using sub-block sizes 128, 256, 512, 1024, and 2048 using Fibonacci encoding to encode the parameters b, k, and the number of exceptions, as well as the exception lists, hence the _Fibonacci_Fibonacci suffix in the table.

4.3 EC compression results

The following table shows the compression ratio achieved for EC column of 7 BUS files ranging from 20-600MB in size.

Table 2: Compression rations of the EC column for various compression methods.

	Compression ratio						
method	0.bus	1.bus	2.bus	3.bus	4.bus	5.bus	6.bus
Increment - EliasDelta	1.462494	1.199965	1.199594	1.463055	1.462947	1.256509	1.256486
Xor - RunLength - Increment - EliasDelta	1.474521	1.272926	1.275875	1.474656	1.474424	1.260942	1.261043
Xor - Increment - EliasDelta	1.475165	1.273768	1.276745	1.475315	1.475070	1.261339	1.261443
Xor - Delta - NegToPosMap - RunLength - Increment - EliasDelta	1.443001	1.202311	1.202463	1.443144	1.443006	1.239592	1.239594
Xor - Delta - NegToPosMap - Increment - EliasDelta	1.443566	1.202651	1.202808	1.443722	1.443570	1.239937	1.239943
Increment - EliasRiceDelta_2	1.534855	1.207108	1.206685	1.535179	1.535124	1.283847	1.283807
Xor - RunLength - Increment - EliasRiceDelta_2	1.544424	1.285990	1.289085	1.544474	1.544294	1.281015	1.281107
Xor - Increment - EliasRiceDelta_2	1.546074	1.288250	1.291434	1.546172	1.545952	1.282062	1.282159
Xor - Delta - NegToPosMap - RunLength - Increment - EliasRiceDelta_2	1.518406	1.211566	1.211682	1.518475	1.518344	1.257091	1.257084
Xor - Delta - NegToPosMap - Increment - EliasRiceDelta_2	1.520156	1.212445	1.212572	1.520270	1.520103	1.258130	1.258129
Increment - EliasRiceDelta_4	1.540622	1.261038	1.260409	1.540954	1.540918	1.355031	1.355002
Xor - RunLength - Increment - EliasRiceDelta_4	1.547088	1.322292	1.325159	1.547016	1.546903	1.355494	1.355585
Xor - Increment - EliasRiceDelta_4	1.549840	1.326270	1.329291	1.549845	1.549662	1.357448	1.357551
Xor - Delta - NegToPosMap - RunLength - Increment - EliasRiceDelta_4	1.520577	1.237942	1.237895	1.520538	1.520462	1.305612	1.305578
Xor - Delta - NegToPosMap - Increment - EliasRiceDelta_4	1.523494	1.239467	1.239442	1.523541	1.523398	1.307480	1.307458
Increment - EliasRiceDelta_8	1.615182	1.297321	1.296886	1.615861	1.615757	1.364360	1.364330
Xor - RunLength - Increment - EliasRiceDelta_8	1.612999	1.361168	1.363790	1.612803	1.612793	1.361516	1.361598
Xor - Increment - EliasRiceDelta_8	1.618382	1.368776	1.371689	1.618335	1.618206	1.365071	1.365173
Xor - Delta - NegToPosMap - RunLength - Increment - EliasRiceDelta_8	1.576089	1.293037	1.293136	1.575898	1.575973	1.336855	1.336825
Xor - Delta - NegToPosMap - Increment - EliasRiceDelta_8	1.581740	1.296038	1.296178	1.581719	1.581672	1.340387	1.340377
Increment - EliasRiceDelta_16	1.882349	1.465610	1.464820	1.882349	1.882349	1.556078	1.555942
Xor - RunLength - Increment - EliasRiceDelta_16	1.868609	1.473411	1.474260	1.868227	1.868537	1.562963	1.562935
Xor - Increment - EliasRiceDelta_16	1.882323	1.490345	1.491800	1.882317	1.882319	1.571836	1.571861
Xor - Delta - NegToPosMap - RunLength - Increment - EliasRiceDelta_16	1.787731	1.416591	1.415978	1.787317	1.787614	1.513006	1.512872
Xor - Delta - NegToPosMap - Increment - EliasRiceDelta_16	1.801522	1.423411	1.422885	1.801520	1.801512	1.521577	1.521492
Increment - EliasRiceDelta_24	1.279998	1.280000	1.280000	1.279998	1.279998	1.280000	1.279999
Xor - RunLength - Increment - EliasRiceDelta_24	1.270664	1.261671	1.261055	1.270405	1.270617	1.271340	1.271292
Xor - Increment - EliasRiceDelta_24	1.279998	1.280000	1.280000	1.279998	1.279998	1.280000	1.279999
Xor - Delta - NegToPosMap - RunLength - Increment - EliasRiceDelta_24	1.269751	1.271888	1.271780	1.269446	1.269673	1.271077	1.271027
Xor - Delta - NegToPosMap - Increment - EliasRiceDelta_24	1.279998	1.280000	1.280000	1.279998	1.279998	1.280000	1.279999
Increment - EliasRiceDelta_32	0.969696	0.969697	0.969697	0.969696	0.969696	0.969697	0.969697
Xor - RunLength - Increment - EliasRiceDelta_32	0.962626	0.955814	0.955347	0.962431	0.962591	0.963139	0.963103
Xor - Increment - EliasRiceDelta_32	0.969696	0.969697	0.969697	0.969696	0.969696	0.969697	0.969697
Xor - Delta - NegToPosMap - RunLength - Increment - EliasRiceDelta_32	0.961935	0.963554	0.963472	0.961704	0.961876	0.962940	0.962902
Xor - Delta - NegToPosMap - Increment - EliasRiceDelta_32	0.969696	0.969697	0.969697	0.969696	0.969696	0.969697	0.969697
Increment - ExpGolomb_2_Unary_Binary	1.197293	0.928346	0.927945	1.197700	1.197625	0.991623	0.991589
Xor - RunLength - Increment - ExpGolomb_2_Unary_Binary	1.203037	0.990634	0.993249	1.203041	1.202902	0.991273	0.991354
Xor - Increment - ExpGolomb_2_Unary_Binary	1.204034	0.991972	0.994640	1.204067	1.203906	0.991901	0.991985
Xor - Delta - NegToPosMap - RunLength - Increment - ExpGolomb_2_Unary_Binary	1.172255	0.921903	0.921950	1.172277	1.172185	0.966512	0.966502
Xor - Delta - NegToPosMap - Increment - ExpGolomb_2_Unary_Binary	1.173296	0.922411	0.922465	1.173347	1.173232	0.967127	0.967120
Increment - ExpGolomb_3_Unary_Binary	1.243830	0.956080	0.955655	1.244268	1.244191	1.023299	1.023264
Xor - RunLength - Increment - ExpGolomb_3_Unary_Binary	1.248588	1.019597	1.022269	1.248565	1.248440	1.022171	1.022253
Xor - Increment - ExpGolomb_3_Unary_Binary	1.250017	1.021487	1.024234	1.250034	1.249877	1.023060	1.023147
Xor - Delta - NegToPosMap - RunLength - Increment - ExpGolomb_3_Unary_Binary	1.215478	0.948435	0.948472	1.215469	1.215395	0.995917	0.995905
Xor - Delta - NegToPosMap - Increment - ExpGolomb_3_Unary_Binary	1.216970	0.949152	0.949198	1.217006	1.216898	0.996788	0.996779
Increment - ExpGolomb_5_Unary_Binary	1.348564	1.016787	1.016307	1.349080	1.348987	1.093077	1.093038
Xor - RunLength - Increment - ExpGolomb_5_Unary_Binary	1.350213	1.082010	1.084757	1.350113	1.350036	1.089977	1.090057
Xor - Increment - ExpGolomb_5_Unary_Binary	1.352725	1.085205	1.088079	1.352696	1.352558	1.091494	1.091583
Xor - Delta - NegToPosMap - RunLength - Increment - ExpGolomb_5_Unary_Binary	1.311773	1.005987	1.005994	1.311688	1.311661	1.060363	1.060338
Xor - Delta - NegToPosMap - Increment - ExpGolomb_5_Unary_Binary	1.314382	1.007197	1.007220	1.314374	1.314284	1.061843	1.061827
Increment - ExpGolomb_8_Unary_Binary	1.540724	1.123583	1.122999	1.541408	1.541274	1.217125	1.217075
Xor - RunLength - Increment - ExpGolomb_8_Unary_Binary	1.532470	1.187460	1.190155	1.532275	1.532286	1.209560	1.209636
Xor - Increment - ExpGolomb_8_Unary_Binary	1.537330	1.193246	1.196166	1.537266	1.537169	1.212366	1.212458
Xor - Delta - NegToPosMap - RunLength - Increment - ExpGolomb_8_Unary_Binary	1.484413	1.105177	1.105084	1.484196	1.484268	1.173705	1.173660
Xor - Delta - NegToPosMap - Increment - ExpGolomb_8_Unary_Binary	1.489426	1.107369	1.107305	1.489358	1.489317	1.176427	1.176397
Increment - ExpGolomb_13_Unary_Binary	1.880204	1.352838	1.352079	1.880660	1.880639	1.481518	1.481452
Xor - RunLength - Increment - ExpGolomb_13_Unary_Binary	1.853652	1.396439	1.398302	1.853369	1.853660	1.460593	1.460612
Xor - Increment - ExpGolomb_13_Unary_Binary	1.864746	1.408934	1.411263	1.864773	1.864805	1.466969	1.467021
Xor - Delta - NegToPosMap - RunLength - Increment - ExpGolomb_13_Unary_Binary	1.799803	1.310418	1.309919	1.799533	1.799788	1.412478	1.412373
Xor - Delta - NegToPosMap - Increment - ExpGolomb_13_Unary_Binary	1.811306	1.315219	1.314781	1.811376	1.811373	1.418621	1.418548
Increment - ExpGolomb_21_Unary_Binary	1.454543	1.454545	1.454545	1.454543	1.454543	1.454545	1.454545
Xor - RunLength - Increment - ExpGolomb_21_Unary_Binary	1.443936	1.433715	1.433015	1.443639	1.443878	1.444702	1.444649
Xor - Increment - ExpGolomb_21_Unary_Binary	1.454543	1.454545	1.454545	1.454543	1.454543	1.454545	1.454545
Xor - Delta - NegToPosMap - RunLength - Increment - ExpGolomb_21_Unary_Binary	1.442898	1.445255	1.445132	1.442549	1.442807	1.444403	1.444346
Xor - Delta - NegToPosMap - Increment - ExpGolomb_21_Unary_Binary	1.454543	1.454457	1.454456	1.454543	1.454543	1.454545	1.454545
Increment - ExpElias_2	1.190902	0.957325	0.956856	1.191157	1.191243	1.031649	1.031594
Xor - RunLength - Increment - ExpElias_2	1.204842	1.025532	1.028020	1.204957	1.204794	1.014351	1.014446
Xor - Increment - ExpElias_2	1.205601	1.026555	1.029081	1.205739	1.205562	1.014826	1.014924
Xor - Delta - NegToPosMap - RunLength - Increment - ExpElias_2	1.191523	0.974050	0.974148	1.191744	1.191582	1.006945	1.006947
Xor - Delta - NegToPosMap - Increment - ExpElias_2	1.192269	0.974462	0.974566	1.192507	1.192329	1.007395	1.007401
Increment - ExpElias_3	1.191976	0.963593	0.963085	1.192238	1.192325	1.043228	1.043165
Xor - RunLength - Increment - ExpElias_3	1.206444	1.030668	1.033176	1.206524	1.206377	1.023309	1.023402
Xor - Increment - ExpElias_3	1.207207	1.031702	1.034248	1.207309	1.207146	1.023792	1.023888
Xor - Delta - NegToPosMap - RunLength - Increment - ExpElias_3	1.192914	0.977558	0.977640	1.193119	1.192961	1.013545	1.013542
Xor - Delta - NegToPosMap - Increment - ExpElias_3	1.193657	0.977973	0.978061	1.193883	1.193710	1.014002	1.014001
Increment - ExpElias_5	1.203649	0.984630	0.984132	1.203904	1.203994	1.054571	1.054517
Xor - RunLength - Increment - ExpElias_5	1.215068	1.051968	1.054492	1.215073	1.214920	1.038661	1.038767
Xor - Increment - ExpElias_5	1.215844	1.053045	1.055608	1.215868	1.215700	1.039159	1.039269
Xor - Delta - NegToPosMap - RunLength - Increment - ExpElias_5	1.200356	0.991704	0.991725	1.200512	1.200361	1.031486	1.031493
Xor - Delta - NegToPosMap - Increment - ExpElias_5	1.201109	0.992131	0.992158	1.201286	1.201121	1.031959	1.031968
Increment - ExpElias_8	1.259366	0.986453	0.985948	1.259902	1.259939	1.058783	1.058732
Xor - RunLength - Increment - ExpElias_8	1.276166	1.057358	1.059939	1.276224	1.276056	1.045847	1.045957
Xor - Increment - ExpElias_8	1.277021	1.058447	1.061067	1.277099	1.276916	1.046351	1.046464
Xor - Delta - NegToPosMap - RunLength - Increment - ExpElias_8	1.249847	1.003533	1.003626	1.250030	1.249911	1.037890	1.037884
Xor - Delta - NegToPosMap - Increment - ExpElias_8	1.250658	1.003971	1.004070	1.250870	1.250733	1.038369	1.038365
Increment - ExpElias_13	1.400850	1.086642	1.086100	1.401229	1.401159	1.157176	1.157102
Xor - RunLength - Increment - ExpElias_13	1.406588	1.161446	1.164157	1.406895	1.406659	1.154426	1.154539
Xor - Increment - ExpElias_13	1.407629	1.162758	1.165518	1.407964	1.407708	1.155041	1.155158
Xor - Delta - NegToPosMap - RunLength - Increment - ExpElias_13	1.366659	1.070492	1.070261	1.366697	1.366598	1.140178	1.140173

Xor - Delta - NegToPosMap - Increment - ExpElias_13	1.367636	1.070989	1.070764	1.367700	1.367581	1.140757	1.140754
Increment - ExpElias_21	1.398575	1.156594	1.156249	1.399089	1.398989	1.209035	1.209014
Xor - RunLength - Increment - ExpElias_21	1.409114	1.223548	1.226249	1.409227	1.409025	1.212827	1.212918
Xor - Increment - ExpElias_21	1.410157	1.225006	1.227759	1.410295	1.410071	1.213506	1.213603
Xor - Delta - NegToPosMap - RunLength - Increment - ExpElias_21	1.380259	1.158505	1.158643	1.380375	1.380256	1.193052	1.193053
Xor - Delta - NegToPosMap - Increment - ExpElias_21	1.381255	1.159087	1.159233	1.381399	1.381259	1.193684	1.193690
Increment - Fibonacci	1.463494	1.157571	1.157027	1.464035	1.463999	1.237922	1.237883
Xor - RunLength - Increment - Fibonacci	1.466258	1.235161	1.238230	1.466381	1.466208	1.234563	1.234663
Xor - Increment - Fibonacci	1.467303	1.236586	1.239709	1.467456	1.467253	1.235230	1.235335
Xor - Delta - NegToPosMap - RunLength - Increment - Fibonacci	1.437816	1.159045	1.159105	1.437943	1.437808	1.210198	1.210186
Xor - Delta - NegToPosMap - Increment - Fibonacci	1.438875	1.159598	1.159666	1.439035	1.438874	1.210843	1.210835
NewPFD_128_Fibonacci_Fibonacci	1.967217	1.577698	1.577664	1.967100	1.967206	1.658945	1.659021
NewPFD_256_Fibonacci_Fibonacci	1.982457	1.587969	1.587967	1.982003	1.982456	1.669278	1.669246
NewPFD_512_Fibonacci_Fibonacci	1.984821	1.588714	1.588697	1.984185	1.985466	1.670386	1.670371
NewPFD_1024_Fibonacci_Fibonacci	1.988689	1.591009	1.591050	1.986672	1.987994	1.673046	1.673299
NewPFD_2048_Fibonacci_Fibonacci	1.990630	1.592162	1.592182	1.988610	1.987252	1.674568	1.674817
raw_size	1.000000	1.000000	1.000000	1.000000	1.000000	1.000000	1.000000
gz1	1.799837	1.677109	1.684655	1.804122	1.802159	1.682700	1.683474
gz9	1.938520	1.789956	1.802806	1.942817	1.940772	1.753917	1.754799

4.4 Description of integer encoders

Elias Delta is defined as

$$\delta(j) = \gamma(|\beta(j)|)\hat{\beta}(j)$$

where $\beta(j)$ is the binary representation of j, $|\cdot|$ represents the number of bits of a codeword, and $\hat{\beta}(j)$ is the headless binary representation of j, i.e. omits the leading 1-bit of β. γ is defined as

$$\gamma(j) = \alpha(|\beta(j)|)\hat{\beta}(j),$$

where $\alpha(j)$ is the unary representation of j. Thus, δ uses γ to code the number of bits needed to code the number, then outputs the bits of the binary representation of j, without the leading 1-bit. Similarly, γ uses α to code the number of bits.

Elias Delta Rice is similar to the Elias Delta (δ) encoding but is parameterized by k. Denote by $j_{n\ldots k+1}$ and $j_{k\ldots 1}$the number where the k least significant bits of j are omitted and the number the k least significant bits code for, respectively. The Elias Delta Rice is then defined by

$$\delta_r(j) = \delta(j_{n\ldots k+1})\beta(j_{k\ldots 1}).$$

In simpler terms, the codec uses δ to encode the number represented by all but the k least significant bits of $\beta(j)$, succeeded by the k least significant bits of $\beta(j)$.

Exponential Golomb encodes an integer x by expressing it as a bucket index H, and an offset R. The bucket index is an index into the following vector of buckets, parameterized by k:

$$B = \left[0, 2^k, \sum_{i=0}^{1} 2^{k+i}, \sum_{i=0}^{2} 2^{k+i}, \sum_{i=0}^{3} 2^{k+i} \ldots\right].$$

H is then chosen such that $B[H] < x \leq B[H+1]$, which is encoded using the Enc_H encoder. R is then computed as the offset $x - B[H]$ and is encoded by the Enc_R encoder. The encoders Enc_H and Enc_R can be chosen arbitrarily.

Exponential Elias encodes an integer x in a similar manner as Exponential Golomb, but uses Elias Delta encoding for both Enc_H and Enc_R.

Huffman encoding Is a known tree-based encoding scheme, assigning each value a prefix-free binary encoding. In order to use this encoder, the compressor must either include the value-to-binary mapping, or use a predefined mapping.
